# LEVERAGING THE NIH TOOLBOX FOR EARLY DETECTION OF COGNITIVE IMPAIRMENT IN PRIMARY CARE: THE TOOLBOX DETECT TRIAL

**DOI:** 10.1093/geroni/igae098.1929

**Published:** 2024-12-31

**Authors:** Michael Wolf, Cindy Nowinski

**Affiliations:** Northwestern University, Evanston, Illinois, United States; Northwestern University, Evanston, Illinois, United States

## Abstract

The NIH Toolbox comprises practice standard, cognitive and neurological functional assessments that can be deployed via iPad for research purposes. Our Northwestern team previously validated two of these brief measures of episodic memory and executive function to have clinical utility. Combined, these scored tests can be mobilized as a primary care-based screening tool for detecting cognitive impairment and dementia. To address common time and workflow constraints in these often-busy clinical settings, our derived assessment, known as Toolbox Detect, was adapted accordingly to be self-administered, and to be tethered to the electronic health record thereby removing manual data entry, followed by clear, ‘turnkey’ recommendations to address any suspected impairment. The effectiveness of Toolbox Detect to improve the timely detection, diagnosis, and referral for cognitive impairment is now being evaluated in a large, pragmatic trial conducted across 40 primary care sites within one academic health system, with real-time findings informing an embedded dissemination and implementation study at 20 federally qualified health centers. Early evidence of the fidelity of the Toolbox Detect strategy’s implementation, in terms of feasibility and acceptability and learnings of how it best might be adapted to community centers serving diverse patient populations will be explored in this presentation. Specific attention will be paid to the challenges and opportunities of introducing new technologies into primary care workflows, and healthcare provider, patient and care partner unmet needs in terms of cognitive screening.

